# A case-control study of necrotising enterocolitis in very low birth weight neonates at a tertiary hospital in South Africa

**DOI:** 10.3389/fped.2026.1878637

**Published:** 2026-09-25

**Authors:** Nkatha Meme, Ajayi Oluseyi Mojisola, Tanusha Ramdin

**Affiliations:** Department of Paediatrics and Child Health, University of the Witwatersrand and Charlotte Maxeke Johannesburg Academic Hospital, School of Clinical Medicine, Faculty of Health Sciences, University of the Witwatersrand, Johannesburg, South Africa

**Keywords:** case control, conditional logistic regression, multiple imputation, necrotizing entercolitis, sepsis, South Africa, very low birth weight (VLBW)

## Abstract

Between January 2018 to June 2020, 119 of 1,106 Very Low Birth Weight (VLBW) neonates admitted to Charlotte Maxeke Johannesburg Academic Hospital (CMJAH) were diagnosed with Necrotising Enterocolitis (NEC), representing 10.8% of the VLBW neonates. This retrospective matched case-control study (119 cases, 119 controls; matched 1:1 on birthweight ±100 g, gestational age ±2 weeks, and postnatal age ±1 week) aimed to determine the prevalence, risk factors, and outcomes of NEC in VLBW neonates at a tertiary hospital in South Africa. We handled missing data using Multiple Imputation by Chained Equations (MICE, M = 20) after confirming the data were Missing at Random. We used MI-pooled conditional logistic regression for all analyses to account for the matched design. The mortality rate among NEC cases was 49.6%. On univariate analysis, outborn status (cOR 3.69, 95% CI 1.84–7.43), conventional ventilation (cOR 4.73, 95% CI 2.52–8.88), late-onset sepsis (cOR 6.29, 95% CI 3.02–13.07), and fungal sepsis (cOR 6.50, 95% CI 2.27–18.62) were significantly associated with NEC. In the multivariable analysis, conventional ventilation (acOR 3.37, 95% CI 1.42–8.01, *p* = 0.006) and late-onset sepsis (acOR 5.27, 95% CI 1.84–15.06, *p* = 0.002) were independently associated with NEC. The final model demonstrated excellent discrimination (AUC = 0.827). Maternal factors were not independently associated with NEC. The prevalence and mortality of NEC among VLBW neonates remain high in this tertiary setting; this aligns with previous studies from the region. Late-onset sepsis and conventional ventilation were the strongest independent risk factors, highlighting the importance of stringent infection prevention and early recognition of clinical deterioration.

## Introduction

Globally, necrotising enterocolitis (NEC) occurs in approximately 2%–5% of all neonates admitted to a neonatal intensive care unit, among whom 5%–10% are very low birth weight (VLBW). Improved interventions and management in neonatal care have translated into an increased survival rate for extremely low and VLBW neonates, who are at increased risk of developing NEC (1–6).

Variability in the incidence and prevalence of NEC has been reported across different parts of the world (4, 7). A study conducted at a South African tertiary neonatal unit compared mortality and morbidity in VLBW neonates between 2006/2007 and 2013 and found that NEC increased from 5.6% to 7.3% (8). Another study from 2013 to 2018 reported a NEC prevalence of 10% (9). A study in the Western Cape in 2013 compared short-term outcomes in inborn and outborn neonates weighing < 1500 g and found the incidence of NEC to be 5.9% among inborn and 8.7% among outborn neonates (10).

Despite improved interventions, including nasal continuous positive airway pressure (NCPAP), antenatal steroids, exogenous surfactant, neonatal intensive care, and ventilator support in tertiary hospitals in South Africa, studies have demonstrated an increase in NEC in their neonatal units (4, 8). A study conducted at a tertiary hospital in South Africa showed that improved survival of VLBW neonates accounted for the reported increase in NEC from 5% to 8% between 2013 and 2017 (11).

The exact pathophysiology of NEC has not been fully elucidated and remains only partially understood (4–6, 12). NEC development is thought to be multifactorial, with several risk factors implicated. The most commonly reported risk factors are low birth weight (LBW) and prematurity, with the highest incidence among LBW and low gestational age (2–5, 11, 12). Depending on severity, treatment of NEC ranges from a conservative approach to surgery involving resection of the affected intestine (4, 10, 13).

Implementing certain interventions to prevent NEC in VLBW neonates has been associated with improved survival. In 2013, NEC prevention was shown to be significantly associated with survival among VLBW neonates in a South African neonatal unit (8). Knowing the current prevalence of NEC and its associated risk factors at the Charlotte Maxeke Johannesburg Academic Hospital (CMJAH) neonatal unit may help identify and develop prevention strategies to support NEC management.

The study aimed to determine the prevalence, associated risk factors and outcome of necrotising enterocolitis in VLBW neonates admitted to the neonatal unit at a tertiary hospital in South Africa from January 2018 to June 2020.

## Materials and methods

### Study design, sampling and setting

This was a retrospective case-control study of VLBW neonates diagnosed with NEC between 1 January 2018 and 30 June 2020 (Figure 1). We conducted a secondary analysis of an existing database at CMJAH. VLBW neonates with NEC at Bell's stage ≥ 2A were selected and classified as cases. Controls were VLBW neonates admitted to the CMJAH neonatal unit with a diagnosis other than NEC. Controls were matched to cases at a 1:1 ratio for birthweight (within 100 g of the case birthweight), gestational age (within 2 weeks of the case gestational age), and postnatal age (within 1 week of the case postnatal age). All controls meeting the 3-variable criteria were placed in a risk pool, and simple random sampling was used to select the final control for each case.

**Figure 1:**
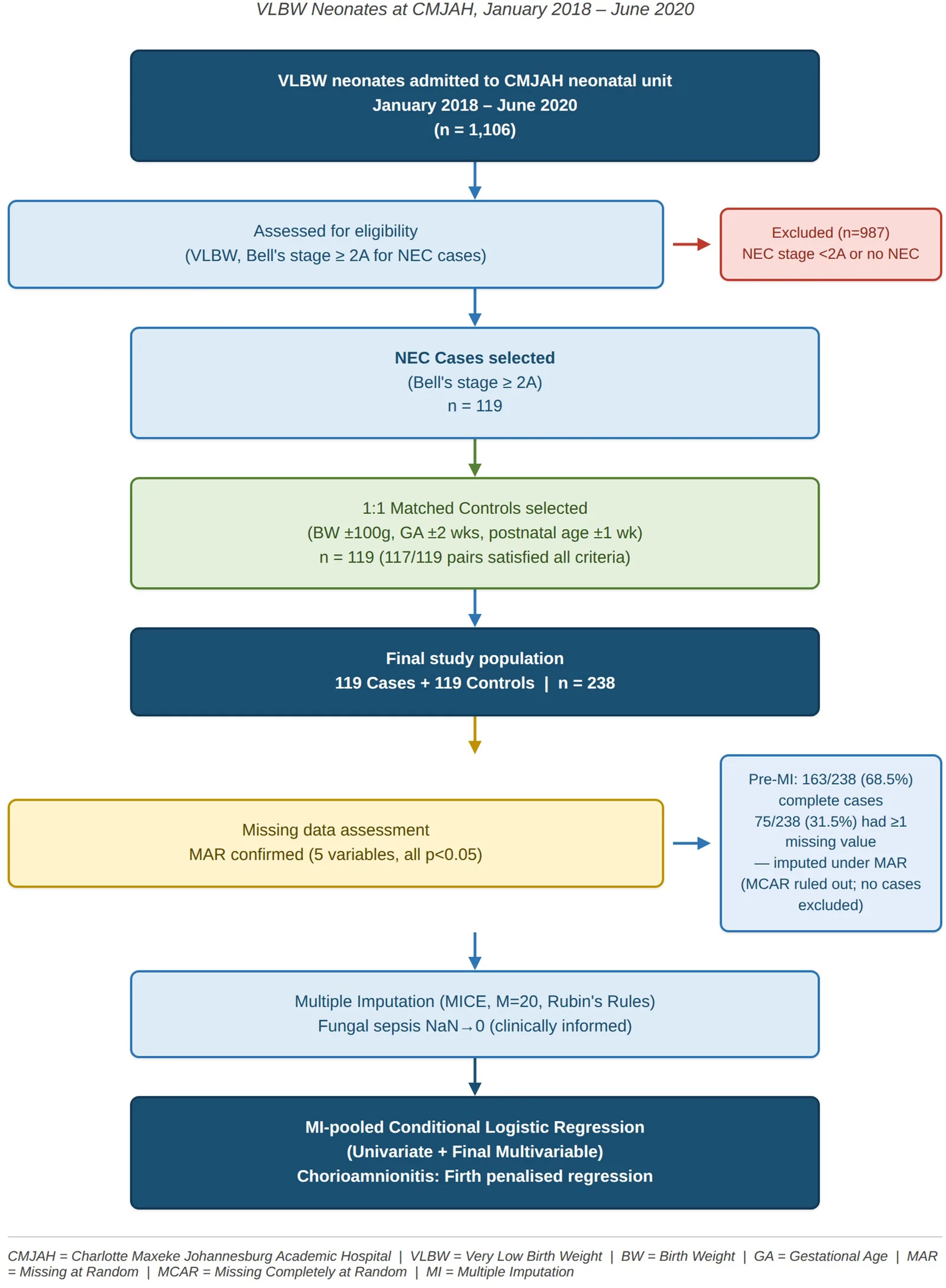
Study Flow Diagram — submitted as separate file.

Charlotte Maxeke Johannesburg Academic Hospital is a tertiary hospital in Parktown, Gauteng Province, South Africa. It is a major referral centre for NEC. The neonatal unit has 90 beds, a 6-bed kangaroo mother care ward, and a 14-bed Intensive Care Unit (ICU) that admits both neonates and paediatric patients. The neonatal unit at CMJAH admits about 1,500 VLBW neonates per year. With an estimated NEC rate of 10%, this translates to about 150 cases of NEC. The estimated sample size was 150 cases and 150 control neonates.

### Data collection and analysis

The neonatal database at CMJAH was reviewed to identify all VLBW neonates with NEC (Bell's stage ≥ 2A) (14) admitted between 1 January 2018 and 30 June 2020. Discharge information was collected and entered into a computer database. These data are managed using Research Electronic Data Capture (REDCap), hosted by the University of the Witwatersrand (15).

The data were extracted from the REDCap database and entered into a prepared data sheet capturing maternal and neonatal characteristics, NEC staging, and outcome. Before analysis, missing data were assessed for all variables. Missingness was significantly associated with NEC status for five variables (Apgar score at 5 min, maternal age, mode of delivery, fungal sepsis, and chorioamnionitis; all *p* < 0.05 by chi-square test), indicating that the data were Missing at Random (MAR) rather than Missing Completely at Random (MCAR). Accordingly, we performed Multiple Imputation by Chained Equations (MICE) across M = 20 imputed datasets using iterated regression with posterior sampling. We recoded fungal sepsis from missing to absent before imputation, as non-documentation in this REDCap database reflected clinical absence rather than unknown status. All analyses were conducted on each imputed dataset, and results were pooled using Rubin's Rules (1987).

Given the 1:1 matched design, we used conditional logistic regression for all univariate and multivariable analyses to account for the matched-pair structure. We screened variables using MI-pooled univariate conditional logistic regression. We entered variables with *p* < 0.20 simultaneously into a final multivariable conditional logistic regression model. We analysed chorioamnionitis separately using Firth-penalised logistic regression with bootstrapped 95% confidence intervals (1,000 iterations) due to complete separation. We treated birth weight and gestational age as matching (design) variables and did not test them as independent predictors. Results are reported as conditional odds ratios (cOR) and adjusted conditional odds ratios (acOR) with 95% confidence intervals (CI). Statistical significance was set at *p* < 0.05. Model discrimination was assessed using the area under the receiver operating characteristic curve (AUC), model fit using the Hosmer–Lemeshow test, and variance explained using the Nagelkerke pseudo-R^2^. All analyses were performed using Python 3.12 (statsmodels 0.14, scikit-learn, scipy).

### Ethical considerations

The proposal for this study was submitted to the University of the Witwatersrand Protocol Review Committee and Human Research Ethics Committee (HREC) for approval (M200484).

### Definitions

Table 1. Vermont Oxford Network. 2026. https://public.vtoxford.org/.

**Table 1:** Vermont Oxford Network definition of terms.

**Term**	**Definitions used in this study**
Vermont Oxford Network definitions	Standard definitions were applied according to the Vermont Oxford Network criteria.
Chorioamnionitis	Premature and/or prolonged rupture of membranes, maternal fever, and foul-smelling liquor.
Necrotising enterocolitis	Bell's stage 2 or 3.
Patent ductus arteriosus	Diagnosed when confirmed on echocardiogram.
Sepsis	Culture-proven bacterial or fungal sepsis only; suspected or clinical sepsis without microbiological confirmation was classified as no sepsis.
Very low birth weight	Birth weight between 1,000 and 1,500 grams.

## Results

### Prevalence of NEC

A total of 1,106 VLBW neonates were admitted to the neonatal unit between January 1, 2018 and June 30, 2020. Among those admitted, 119 neonates were diagnosed with NEC and fulfilled the inclusion criteria, giving a NEC prevalence of 10.8% during the study period.

### Demographic and clinical characteristics of neonates

Among neonates diagnosed with NEC, the majority (57.1%) were male, and the majority (63.0%) were born in CMJAH (inborn). The majority required conventional ventilation (59.7%), received a blood transfusion (74.8%), developed an infection after day 3 of life (68.9%), and had fungal sepsis (21.8%). NEC surgery was performed in 37 cases (31.1%). The mortality rate was 49.6% (59/119) among cases and 27.7% (33/119) among controls. Tables 2, 3 present the full demographic and clinical characteristics.

**Table 2:** Demographic and clinical characteristics of VLBW neonates with and without NEC at a tertiary hospital in South Africa.

Neonatal Characteristic	Cases (*n* = 119) n (%)	Controls (*n* = 119) n (%)	*p*-value
Birth Characteristics			
Birth Weight (g) — median (IQR)	1,140 (952–1,300)	1,100 (970–1,300)	0.989
1,000–1,499g	79 (66.4%)	83 (69.7%)	
750–999g	34 (28.6%)	32 (26.9%)	
< 750g	5 (4.2%)	4 (3.4%)	
Gestational Age (weeks) — median (IQR)	29 (28–31)	29 (28–30)	0.962
≥ 32 weeks	22 (18.5%)	17 (14.3%)	
28–31 weeks	72 (60.5%)	86 (72.3%)	
< 28 weeks	24 (20.2%)	16 (13.4%)	
Demographics			
Gender: Male	68 (57.1%)	52 (43.7%)	0.069
Place of Birth: Inborn	**75** (**63.0%)**	**102** (**85.7%)**	**<0**.001*
Place of Birth: Outborn	**43** (**36.1%)**	**15** (**12.6%)**	**<0**.001*
Apgar Score at 5 min — median (IQR)	8 (7–9)	9 (7–9)	0.291
Respiratory Support			
Nasal CPAP	71 (59.7%)	79 (66.4%)	1.000
Conventional Ventilation	**71** (**59.7%)**	**29** (**24.4%)**	**<0**.001*
Surfactant Therapy	68 (57.1%)	88 (73.9%)	0.433
Clinical Events			
Blood Transfusion	**89** (**74.8%)**	**67** (**56.3%)**	**0**.001*
Patent Ductus Arteriosus	**22** (**18.5%)**	**11** (**9.2%)**	**0**.047*
Infection ≤ Day 3 (Early Sepsis)	7 (5.9%)	10 (8.4%)	0.725
Infection > Day 3 (Late-onset Sepsis)	**82** (**68.9%)**	**36** (**30.3%)**	**<0**.001*
Fungal Sepsis	**26** (**21.8%)**	**4** (**3.4%)**	**0**.039*
NEC Surgery	**37** (**31.1%)**	**0** (**0.0%)**	**<0**.001*
Outcome			
Died	**59** (**49.6%)**	**33** (**27.7%)**	**<0**.001*
Discharged home	**44** (**37.0%)**	**76** (**63.9%)**	**<0**.001*
Transferred to another hospital	**16** (**13.4%)**	**10** (**8.4%)**	**<0**.001*

*p < 0.05; IQR, interquartile Range; CPAP, continuous positive airway pressure. *p*-values from chi-square test (categorical) or Mann–Whitney U test (continuous). ‘Not Known' responses excluded from *p*-value calculations.

Bold values with * mean the *p* value is < 0.05 and statistically significant.

**Table 3:** Maternal demographic and clinical characteristics of VLBW neonates with and without NEC at a tertiary hospital in South Africa.

Maternal Characteristic	Cases (*n* = 119) n (%)	Controls (*n* = 119) n (%)	*p*-value
Mean Maternal Age (years) — mean (SD)	28.7 (6.2)	30.2 (6.4)	0.134
Mode of Delivery: Normal vaginal delivery	32 (26.9%)	40 (33.6%)	0.181
Mode of Delivery: Caesarean section	59 (49.6%)	75 (63.0%)	0.181
Multiple Gestation	27 (22.7%)	16 (13.4%)	0.055
Attended Antenatal Care	80 (67.2%)	91 (76.5%)	0.196
Antenatal Steroids Given	46 (38.7%)	55 (46.2%)	0.796
Pregnancy-Induced Hypertension	26 (21.8%)	34 (28.6%)	0.739
Maternal HIV Positive	40 (33.6%)	33 (27.7%)	0.261
Chorioamnionitis	**5** (**4.2%)**	**0** (**0.0%)**	**0**.047*

*p < 0.05. ‘Not Known' responses excluded from *p*-value calculations. NVD, normal vaginal delivery.

Bold values with * mean the *p* value is < 0.05 and statistically significant.

### Neonatal clinical and demographic characteristics associated with NEC

On MI-pooled univariate conditional logistic regression, the following variables were significantly associated with NEC (*p* < 0.05): male sex (cOR 1.75, 95% CI 1.03–3.00, *p* = 0.040), outborn status (cOR 3.69, 95% CI 1.84–7.43, *p* < 0.001), multiple gestation (cOR 2.21, 95% CI 1.06–4.60, *p* = 0.035), conventional ventilation (cOR 4.73, 95% CI 2.52–8.88, *p* < 0.001), blood transfusion (cOR 3.06, 95% CI 1.56–5.99, *p* = 0.001), patent ductus arteriosus (cOR 2.64, 95% CI 1.11–6.33, *p* = 0.029), late-onset sepsis (cOR 6.29, 95% CI 3.02–13.07, *p* < 0.001), and fungal sepsis (cOR 6.50, 95% CI 2.27–18.62, *p* < 0.001). Chorioamnionitis, assessed using Firth penalised regression, showed a strong but non-significant association (OR 10.80, 95% CI 1.33–23.20, *p* = 0.112) due to sparse events. Full results are in Table 4.

**Table 4:** Univariate and multivariable conditional logistic regression of factors associated with NEC (MI-pooled, M = 20 imputations, rubin's rules).

Variable	N (pairs)	cOR	95% CI (Uni)	*p* (Uni)	acOR	95% CI (Multi)	*p* (Multi)
Neonatal Demographics							
Gender (Male vs Female)	**119**	**1**.**75**	**1.03**–**3.00**	**0**.040*	**1.64**	**0.69–3.89**	**0.265**
Place of Birth (Outborn vs Inborn)	**119**	**3**.**69**	**1.84**–**7.43**	**<0**.001*	**3.50**	**1.10–11.11**	**0.034** *
Apgar Score at 5 min	119	0.89	0.75–1.04	0.151	0.90	0.71–1.15	0.417
Maternal Characteristics							
Maternal Age (years)	119	0.97	0.93–1.02	0.243	—	—	—
Mode of Delivery (C-section)	119	0.88	0.51–1.51	0.636	—	—	—
Attended ANC	119	1.62	0.77–3.45	0.206	—	—	—
Antenatal Steroids	119	1.11	0.63–1.96	0.708	—	—	—
Maternal Hypertension	119	0.88	0.47–1.63	0.684	—	—	—
Multiple Gestation	**119**	**2**.**21**	**1.06**–**4.60**	**0**.035*	**2.86**	**0.90–9.14**	**0.076**
Maternal HIV	119	1.45	0.84–2.50	0.179	1.81	0.72–4.52	0.205
Neonatal Clinical							
Conventional Ventilation	**119**	**4**.**73**	**2.52**–**8.88**	**<0**.001*	**3.37**	**1.42–8.01**	**0.006** *
Nasal CPAP	119	1.04	0.56–1.94	0.903	—	—	—
Surfactant Therapy	119	0.82	0.42–1.60	0.558	—	—	—
Blood Transfusion	**119**	**3**.**06**	**1.56**–**5.99**	**0**.001*	**2.12**	**0.75–6.01**	**0.156**
PDA (Patent Ductus Arteriosus)	**119**	**2**.**64**	**1.11**–**6.33**	**0**.029*	**1.53**	**0.36–6.47**	**0.567**
Infection ≤ Day 3	119	0.80	0.30–2.14	0.661	—	—	—
Late-onset Sepsis (>Day 3)	**119**	**6**.**29**	**3.02**–**13.07**	**<0**.001*	**5.27**	**1.84–15.06**	**0.002** *
Fungal Sepsis	**119**	**6**.**50**	**2.27**–**18.62**	**<0**.001*	**2.51**	**0.62–10.23**	**0.200**
Chorioamnionitis† (Firth penalised — complete separation)							
Chorioamnionitis†	119	10.80	1.33–23.20	0.112	—	—	— (excluded)

* *p* < 0.05 (highlighted). cOR, conditional Odds Ratio; acOR, adjusted conditional Odds Ratio; 95% CI,  95% Confidence Interval. Estimates were pooled across M = 20 imputed datasets using Rubin's Rules. Variables with *p* < 0.20 in univariate analysis were entered into the multivariable model simultaneously. †Chorioamnionitis: Firth L1-penalised logistic regression, bootstrapped 95% CI (1,000 iterations); excluded from multivariable model (complete separation). Model diagnostics: AUC=0.827, Nagelkerke R^2^ = 0.415, Hosmer–Lemeshow *χ*^2^ = 8.50 (df = 8), *p* = 0.386. uOR, unadjusted Odds Ratios, aOR, adjusted Odds Ratios.

### Maternal clinical characteristics associated with NEC

Overall, maternal characteristics did not show independent significant associations with NEC on multivariable analysis. Multiple gestation (cOR 2.21, *p* = 0.035) and chorioamnionitis (Firth OR 10.80, *p* = 0.112) showed univariate associations; however, these were not retained after multivariable adjustment. These findings are consistent with previously published South African studies (9, 11).

### Neonatal clinical characteristics associated with NEC

On the final MI-pooled multivariable conditional logistic regression model, conventional ventilation (acOR 3.37, 95% CI 1.42–8.01, *p* = 0.006) and late-onset sepsis (acOR 5.27, 95% CI 1.84–15.06, *p* = 0.002) were independently associated with NEC. Outborn status showed a borderline independent association (acOR 3.50, 95% CI 1.10–11.11, *p* = 0.034). The final model, Figure 2 demonstrated excellent discrimination (AUC=0.827, Nagelkerke R^2^ = 0.415) and adequate fit (Hosmer–Lemeshow *χ*^2^ = 8.50, df = 8, *p* = 0.386). Table 4 presents the full results.

**Figure 2:**
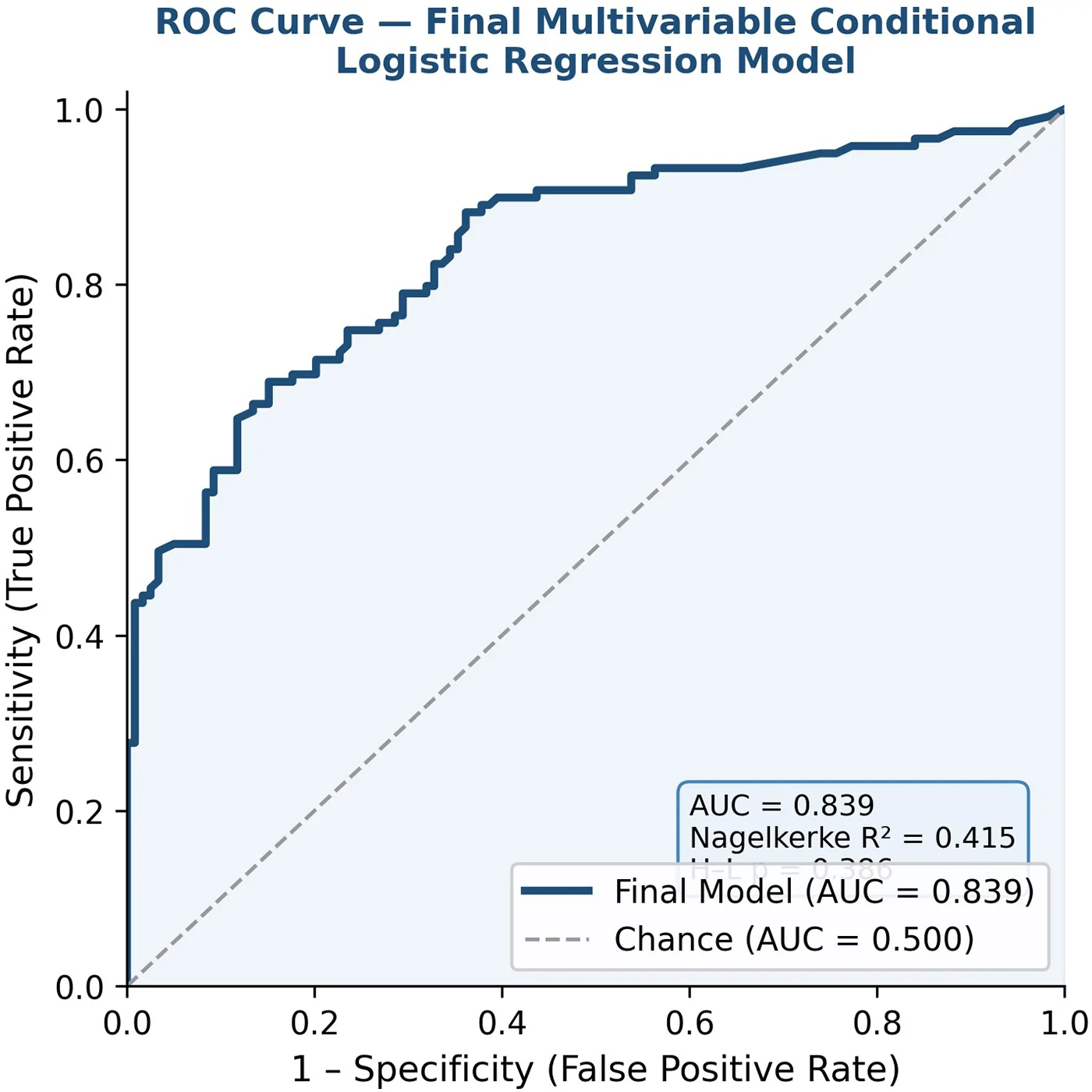
ROC Curve (AUC=0.827) — submitted as separate file.

## Discussion

### Prevalence of NEC

In this study, the prevalence of NEC among VLBW neonates admitted to CMJAH was 10.8%. This finding is comparable to that reported by Ajayi et al. (9) and suggests a continuing rise in NEC prevalence at CMJAH over time. Previous local studies reported NEC rates of 8.2% between 2013 and 2017 (11) and an increase from 5.6% in 2006/2007 to 7.3% in 2013 among VLBW neonates (8). Similar increases have also been reported in high-income countries, including Canada and Denmark (16, 17).

The observed increase may partly reflect improved neonatal interventions, including NCPAP, antenatal corticosteroids, ventilatory support and exogenous surfactant. These advances have improved survival among VLBW neonates, who remain at high risk of NEC and may therefore lead to more NEC diagnoses over time (5, 8, 9, 11).

Charlotte Maxeke Johannesburg Academic Hospital is a tertiary referral hospital that receives a high proportion of premature and clinically complex neonates. This may introduce referral bias and contribute to the higher observed prevalence of NEC. For this reason, comparisons with district-level hospitals or with international settings should be interpreted cautiously.

### Factors associated with NEC

#### Demographic characteristics

Male neonates accounted for 57.1% of NEC cases. Male sex was associated with NEC on univariate analysis (cOR 1.75, *p* = 0.040), but this association was not retained in the multivariable model (acOR 1.64, *p* = 0.265). Previous studies have reported inconsistent findings, with some identifying female sex as a risk factor (11) and others reporting a higher risk among males (4, 18). These differences suggest that biological sex alone has limited predictive value once other clinical factors are considered.

Outborn status was significantly associated with NEC in both univariate (cOR 3.69, *p* < 0.001) and multivariable analyses (acOR 3.50, 95% CI 1.10–11.11, *p* = 0.034). Outborn neonates may be clinically unstable at presentation and may have had reduced access to antenatal care, antenatal corticosteroids and timely perinatal intervention, consistent with findings by Gibbs et al. (10). This finding supports the importance of timely in-utero transfer and effective perinatal regionalisation.

#### Ventilation support and illness severity

Conventional ventilation was independently associated with NEC in both univariate and multivariable analyses (cOR 4.73; acOR 3.37, 95% CI 1.42–8.01, *p* = 0.006).

This finding should be interpreted as a marker of illness severity rather than evidence of causality. Critically ill neonates with NEC may require respiratory support because of haemodynamic instability, infection and systemic inflammation. This is consistent with South African studies showing that conventional ventilation is more common among severely ill neonates with NEC (4, 5, 11).

#### Late-onset sepsis and fungal sepsis

Late-onset sepsis was among the strongest factors associated with NEC. This is biologically plausible, as the immature gastrointestinal and immune systems of preterm neonates increase susceptibility to infection and intestinal inflammation (2, 9).

Infection after day 3 of life was strongly associated with NEC on univariate analysis (cOR 6.29, *p* < 0.001) and remained independently associated after adjustment (acOR 5.27, 95% CI 1.84–15.06, *p* = 0.002).

Fungal sepsis was also associated with NEC on univariate analysis (cOR 6.50, *p* < 0.001), consistent with local studies linking fungal infection and late-onset sepsis to NEC (5, 11, 19). However, it was not independently significant after adjustment (acOR 2.51, *p* = 0.200), suggesting overlap with late-onset sepsis, ventilation and overall illness severity.

In this setting, prolonged hospitalisation, invasive procedures, central line use and prolonged antibiotic exposure may increase the risk of sepsis in VLBW neonates. Because temporality could not be established, these findings support stronger infection-prevention practices rather than causal conclusions.

#### Feeding practices, patent ductus arteriosus (PDA) and blood transfusions

Formula feeding was not associated with NEC in this study, unlike several previous studies (2–4, 9). This finding should be interpreted cautiously, as feeding type, timing and practices before NEC onset were not captured.

PDA was associated with NEC on univariate analysis (cOR 2.64, *p* = 0.029), but not after adjustment (acOR 1.53, *p* = 0.567). Although previous studies have reported PDA as a risk factor (5, 9), the present findings suggest that PDA alone may not independently predict NEC.

Blood transfusion was significant on univariate analysis (cOR 3.06, *p* = 0.001) but not after adjustment (acOR 2.12, *p* = 0.156). Although transfusion-associated NEC has been described (9, 20, 21), evidence remains conflicting (3, 4, 22), and this association may reflect anaemia or illness severity rather than transfusion itself.

Other clinical variables, including respiratory distress at birth, surfactant use, nasal CPAP, feeding type at discharge, and steroid treatment for chronic lung disease and early infection, were not independently associated with NEC.

### Maternal factors

Chorioamnionitis showed a strong but non-significant association with NEC (OR 10.80, 95% CI 1.33–23.20, *p* = 0.112), consistent with Ajayi et al. (9). It could not be included in the multivariable model because all five events occurred in cases, resulting in complete separation and a wide confidence interval.

Maternal HIV exposure, antenatal steroid use and pregnancy-induced hypertension were not associated with NEC, consistent with the findings of Motsisim et al. (11).

### Mortality rate

Mortality among NEC cases was high at 49.6%, comparable with local studies reporting mortality rates of 43.9%–49.7% (4, 8, 9). This likely reflects the combined effects of prematurity, infection severity, delayed referral, limited resources, and restricted postoperative care capacity.

Surgical NEC is known to carry higher mortality than medically managed NEC (5), probably because these neonates are more severely ill. This study did not compare mortality between the surgical and medical NEC groups.

### Study limitations

This retrospective study used secondary database data, so incomplete capture and missing data were unavoidable. Multiple imputation was used after missingness was assumed to be Missing at Random, but residual bias cannot be excluded. The design also limited causal inference, particularly the ability to establish whether sepsis preceded, followed or occurred concurrently with NEC.

There is potential overmatching bias, as GA and birth weight are intrinsically linked to the pathogenesis of NEC. Matching limited our ability to evaluate their independent effects by reducing variability between the cases and controls and potentially masking a true association between NEC and these two known risk factors. Sparse events for variables such as chorioamnionitis and fungal sepsis may also have introduced estimation bias.

The diagnosis of NEC relied on REDCap documentation, and abdominal radiographs could not be reviewed to confirm pneumatosis. Key variables, including duration of ventilation, antibiotic exposure, laboratory results, illness severity scores and central line use, were unavailable.

The database did not include feeds type and duration therefore precluding the assessment of feeding practices prior to the onset of NEC. Post-discharge outcomes were not assessed.

### Study conclusion and recommendations

This study found a NEC prevalence of 10.8% among VLBW neonates at CMJAH, consistent with previous South African studies and suggesting a rising burden over time. Conventional ventilation and late-onset sepsis were independently associated with NEC, whereas fungal sepsis appeared to reflect overlapping illness severity. These findings emphasise the importance of infection prevention, early recognition of late-onset sepsis and timely management of clinical deterioration.

Mortality from NEC remains high in this tertiary South African setting. Prospective studies are needed to clarify exposure timelines, feeding practices and potentially modifiable risk factors for NEC in VLBW neonates.

## Data Availability

The raw data supporting the conclusions of this article will be made available by the authors, without undue reservation.

## References

[1] Muller MJ Paul T Seeliger S. Necrotising enterocolitis in premature infants and newborns. J Neonatal-Perinat Med. (2016) 9(3):233–42. 10.3233/NPM-16915130 27589549

[2] Lin L Xia X Liu W Wang Y Hua Z. Clinical characteristics of neonatal fulminant necrotizing enterocolitis in a tertiary Children's Hospital in the last 10 years. PLoS One. (2019) 14(11):e0224880. 10.1371/journal.pone.0224880 31703096 PMC6839848

[3] Berkhout DJC Klaassen P Niemarkt HJ De Boode WP Cossey V Van Goudoever JB. Risk factors for necrotizing enterocolitis: a prospective multicenter case-control study. Neonatology. (2018) 114(3):277–84. 10.1159/000489677 29996136

[4] Kamanga NHB Thomas R Thandrayen K Velaphi SC. Risk factors and outcomes of infants with necrotizing enterocolitis: a case-control study. Front Pediatr. (2025) 13:1611111. 10.3389/fped.2025.1611111 40994528 PMC12456917

[5] Selse M Saggers RT Hentz E Elfvin A Ballot DE. Outcome of infants with necrotising enterocolitis at charlotte maxeke Johannesburg academic hospital, South Africa. South Afr J Child Health. (2023) 21:9–14. 10.7196/SAJCH.2023.v17i1.1939

[6] Satardien M Van Wyk L Sidler D Van Zyl JI. Outcomes of neonates requiring neonatal intensive care admission for necrotizing enterocolitis in a resource-restricted hospital in Cape Town, South Africa. J Trop Pediatr. (2021) 67(1):fmaa130. 10.1093/tropej/fmaa130 33693891

[7] Alsaied A Islam N Thalib L. Global incidence of necrotizing enterocolitis: a systematic review and meta-analysis. BMC Pediatr. (2020) 20(1):344. 10.1186/s12887-020-02231-5 32660457 PMC7359006

[8] Ballot DE Chirwa T Ramdin T Chirwa L Mare I Davies VA. Comparison of morbidity and mortality of very low birth weight infants in a central hospital in Johannesburg between 2006/2007 and 2013. BMC Pediatr. (2015) 15:20. 10.1186/S12887-015-0337-4 25885283 PMC4373031

[9] Ajayi O Chirwa T Segal B Ballot D. 6289 The incidence and determinants of necrotising enterocolitis among neonates at a public hospital in South Africa (2013–2018). Arch Dis Child. (2024) 109(Suppl 1):A136–7. 10.1136/archdischild-2024-rcpch.200

[10] Gibbs L Tooke L Harrison MC. Short-term outcomes of inborn v. Outborn very-low-birth-weight neonates (<1 500 g) in the neonatal nursery at groote schuur hospital, Cape Town, South Africa. S Afr Med J. (2017) 107(10):900. 10.7196/SAMJ.2017.v107i10.12463 29022536

[11] Motsisim S Ballot D. A review of necrotising enterocolitis in very low birth weight babies in a tertiary hospital in Johannesburg. Afr J Paediatr Surg. (2023) 20(1):59–66. 10.4103/ajps.ajps_156_21 36722571 PMC10117028

[12] Alganabi M Lee C Bindi E Li B Pierro A. Recent advances in understanding necrotizing enterocolitis. F1000Res. (2019) 8:8. 10.12688/F1000RESEARCH.17228.1 30740215 PMC6348433

[13] Lambert DK Christensen RD Baer VL Henry E Gordon PV Besner GE. Fulminant necrotizing enterocolitis in a multihospital healthcare system. J Perinatol. (2012) 32(3):194–8. 10.1038/jp.2011.61 21566569

[14] Kliegman RM Walsh MC. Neonatal necrotizing enterocolitis: pathogenesis, classification, and spectrum of illness. Curr Probl Pediatr. (1987) 17(4):213–88. 10.1016/0045-9380(87)90031-4 3556038 PMC7130819

[15] Harris PA Taylor R Thielke R Payne J Gonzalez N Conde JG. Research electronic data capture (REDCap)––a metadata-driven methodology and workflow process for providing translational research informatics support. J Biomed Inform. (2009) 42(2):377–81. 10.1016/j.jbi.2008.08.010 18929686 PMC2700030

[16] Zani A Pierro A. Necrotizing enterocolitis: controversies and challenges. F1000Res. (2015) 4:F1000 Faculty Rev–1373. 10.12688/f1000research.6888.1 26918125 PMC4753995

[17] Juhl SM Gregersen R Lange T Greisen G. Incidence and risk of necrotizing enterocolitis in Denmark from 1994 to 2014. PLoS One. (2019) 14(7):e0219268. 10.1371/journal.pone.0219268 31283781 PMC6613693

[18] Angura P Velaphi S. Risk factors for necrotising enterocolitis in an HIV-endemic region. Paediatr Int Child Health. (2014) 34(3):208–15. 10.1179/2046905514Y.0000000126 24804768

[19] Ramdin TD Chibabhai V Saggers RT Bandini RM Ballot DE. Epidemiology, risk factors and outcomes associated with candidaemia in very low birth weight infants at a tertiary South African hospital over a 7-year period (2013–2019). Clin Epidemiol Glob Health. (2023) 20:101247. 10.1016/j.cegh.2023.101247

[20] Patel RM Knezevic A Shenvi N Hinkes M Keene S Roback JD. Association of red blood cell transfusion, Anemia, and necrotizing enterocolitis in very low-birth-weight infants. JAMA. (2016) 315(9):889–97. 10.1001/jama.2016.1204 26934258 PMC4805423

[21] Maheshwari A Patel RM Christensen RD. Anemia, red blood cell transfusions, and necrotizing enterocolitis. Necrotizing Enterocolitis. (2018) 27(1):47–51. 10.1053/j.sempedsurg.2017.11.009 PMC5776697 29275817

[22] Dang D Gu X Jiang S Li W Zhou W Cao Y. RBC Transfusion and necrotizing enterocolitis in very preterm infants: a multicenter observational study. Sci Rep. (2024) 14(1):14345. 10.1038/s41598-024-64923-7 38906930 PMC11192881

